# Ascorbic Acid 2-Phosphate Releasing Supercritically Foamed Porous Poly-L-Lactide-Co-ε-Caprolactone Scaffold Enhances the Collagen Production of Human Vaginal Stromal Cells: A New Approach for Vaginal Tissue Engineering

**DOI:** 10.1007/s13770-023-00603-3

**Published:** 2023-10-31

**Authors:** Reetta Sartoneva, Kaarlo Paakinaho, Markus Hannula, Kirsi Kuismanen, Heini Huhtala, Jari Hyttinen, Susanna Miettinen

**Affiliations:** 1 Faculty of Medicine and Health Technology (MET), Tampere University, Arvo Ylpön Katu 34, 33520 Tampere, Finland; 2 Tays Research Services, Wellbeing Services County of Pirkanmaa, Tampere University Hospital, Arvo Ylpön Katu 34, 33520 Tampere, Finland; 3 Department of Obstetrics and Gynaecology, Seinäjoki Central Hospital, Seinäjoki, Finland; 4 Department of Obstetrics and Gynaecology, Tampere University Hospital, Tampere, Finland; 5 Faculty of Social Sciences, University of Tampere, Tampere, Finland

**Keywords:** Poly-L-lactide-co-ε-caprolactone, Porous scaffold, Ascorbic acid 2-phosphate, Vaginal tissue engineering, Vaginal epithelial cell, Vaginal stromal cell

## Abstract

**Background:**

The reconstructive surgery of vaginal defects is highly demanding and susceptible to complications, especially in larger defects requiring nonvaginal tissue grafts. Thus, tissue engineering-based solutions could provide a potential approach to the reconstruction of vaginal defects.

**Methods:**

Here, we evaluated a novel porous ascorbic acid 2-phosphate (A2P)-releasing supercritical carbon dioxide foamed poly-L-lactide-co-ε-caprolactone (scPLCL_A2P_) scaffold for vaginal reconstruction with vaginal epithelial (EC) and stromal (SC) cells. The viability, proliferation, and phenotype of ECs and SCs were evaluated in monocultures and in cocultures on d 1, d 7 and d 14. Furthermore, the collagen production of SCs on scPLCL_A2P_ was compared to that on scPLCL without A2P on d 14.

**Results:**

Both ECs and SCs maintained their viability on the scPLCL_A2P_ scaffold in mono- and coculture conditions, and the cells maintained their typical morphology during the 14-d culture period. Most importantly, the scPLCL_A2P_ scaffolds supported the collagen production of SCs superior to plain scPLCL based on total collagen amount, collagen I and III gene expression results and collagen immunostaining results.

**Conclusion:**

This is the first study evaluating the effect of A2P on vaginal tissue engineering, and the results are highly encouraging, indicating that scPLCL_A2P_ has potential as a scaffold for vaginal tissue engineering.

**Graphical Abstract:**

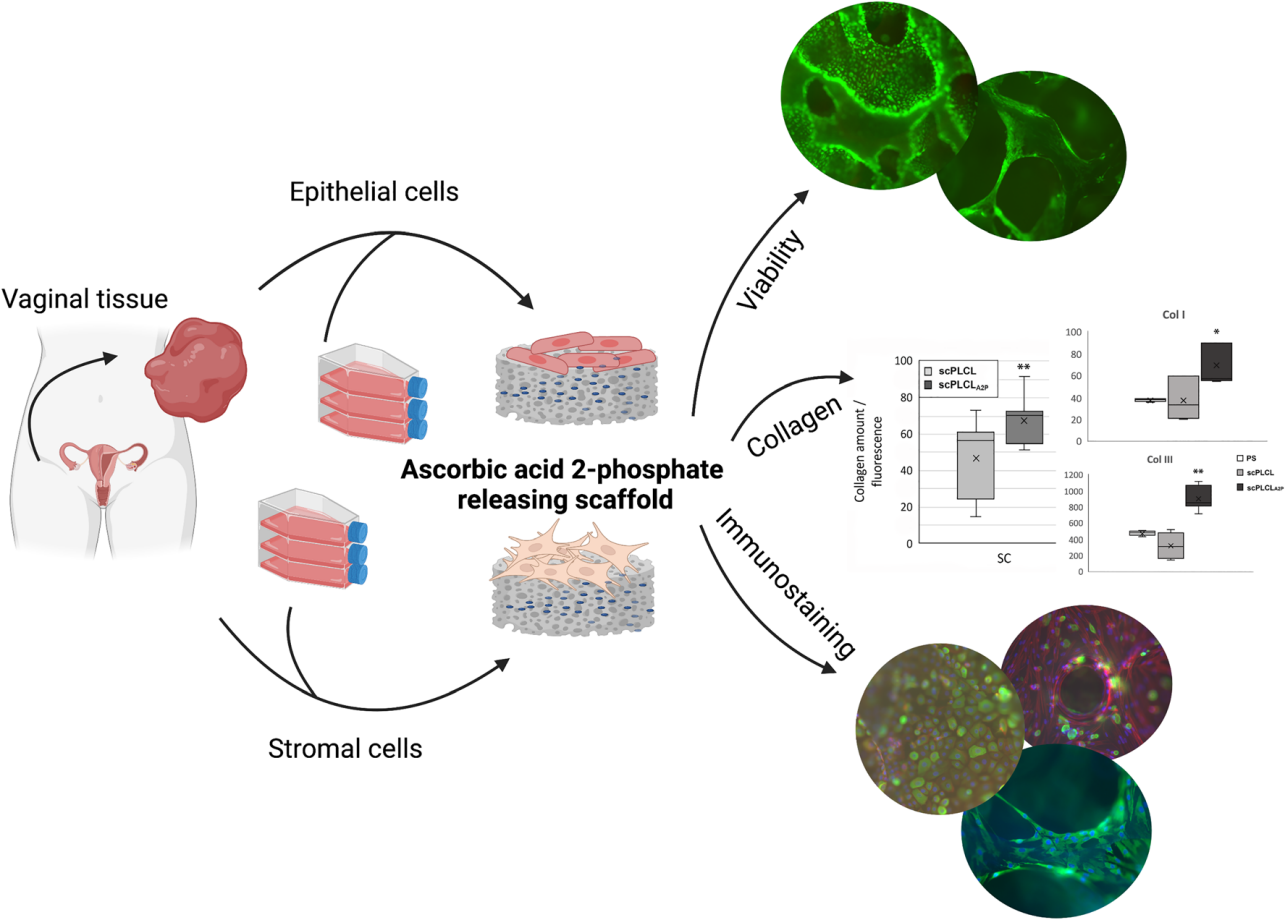

## Introduction

The causes for vaginal defects requiring reconstruction are multifactorial. Mayer–Rokitanky–Kuster–Hauser (MRKH) is a congenital disorder in which the development of internal genitalia is disturbed [1, 2]. Operative surgery for cancer or pelvic organ prolapse may require extirpation of vaginal tissue and cause shortening of the vagina. Additionally, transgender surgery is an emerging and challenging field in reconstructive surgery. In general, the first-line treatment for vaginal defects is dilatation; however, it requires notable patience, and the success rate is limited [3]. Reconstructive surgery is a second-line treatment option, and various nonvaginal tissues, such as skin grafts, intestine, peritoneum, or amniotic membrane, have been used for vaginal reconstruction. However, the use of nonvaginal acellular tissue grafts may cause problems such as stenosis, graft shrinkage, lack of lubrication and mucous secretion. Furthermore, the use of nonvaginal grafts may increase the risk of de novo malignancy [4–6].

Tissue engineering combining cells, biomaterials, and active agents could be an interesting option for the surgical reconstruction of vaginal defects in the future. However, research in this field is still very limited. The selection of an appropriate biomaterial is crucial, and the scaffold material should be biodegradable, biocompatible, easy to handle and suture, easy to form into a tubular structure, and most importantly, able to facilitate the regeneration of normal vaginal histology. The advantages of synthetic biomaterials over natural biomaterials are that synthetic biomaterials can be manufactured on a large scale with consistent quality, and the mechanical properties and scaffold design can be tailored according to the requirements for the reconstructed tissue [6–9]. Previously, De Filippo et al. published results of total vaginoplasty in a rabbit model regenerated with either a tissue-engineered poly-lactide-co-glycolide-coated polyglycolic acid scaffold cultured with vaginal epithelial and smooth muscle cells or an unseeded control scaffold. In the control group, vaginal stricture or graft collapse was evident one month after surgery, and after three months, a thin fibrotic scar was detected. In contrast, in the cell-seeded tissue-engineered scaffold group after six months, normal vaginal histology was detected, and the tissue-engineered graft maintained the normal vaginal caliber [7]. Additionally, poly-l-lactide-co-ε-caprolactone (PLCL) is a widely studied biomaterial that meets all the above-mentioned criteria and is especially favored for soft tissue engineering applications such as urothelial, vaginal, neural, and vascular tissue engineering due to its flexibility and softness [10–16]. Numerous fabrication methods have been used to produce porous 3D scaffolds for tissue engineering applications; however, many of these methods, such as salt leaching and electrospinning, require solvents that might be toxic. Supercritical carbon dioxide (CO_2_) foaming allows the manufacture of porous 3D scaffolds without any toxic solvents, which is highly favorable when developing scaffolds for clinical applications [16–19]. Previously, the supercritical CO_2_ foamed PLCL (scPLCL) scaffolds were shown to support the viability and proliferation of human vaginal epithelial cells (ECs) and human vaginal stromal cells (SCs) [16]. However, some degree of cell clustering was detected with plain scPLCL, which might be due to the hydrophobicity of plain PLCL [20, 21].

Ascorbic acid (AA) is a nontoxic natural compound known as vitamin C, which has been shown to have a crucial role in collagen synthesis. Furthermore, supplementing cell culture medium with AA has been shown to facilitate stromal cell proliferation, collagen production and stem cell differentiation [22–24]. However, the stability of AA in* in vitro* culture conditions is low, and therefore, finding new techniques to utilize AA’s effects on cells could be useful in reconstructive tissue engineering applications. Ascorbic acid 2-phosphate (A2P), a more stable form of AA, has attracted interest in tissue engineering [23, 25–28]. The utility of A2P has been especially recognized in bone tissue engineering; however, in gynecological applications, increased collagen production is highly desirable [23, 26, 29–31]. Embedding AA or its derivatives (AAD) directly into the scaffold has emerged as an interesting idea for tissue engineering, allowing AA release from the scaffold [17, 26]. Previously, A2P-embedded porous polylactide (PLA) and PLCL scaffolds have been studied for pelvic organ prolapse and urological applications with potentially encouraging results. Based on the results, A2P enhances the proliferation of stromal cells; furthermore, A2P increases the hydrophilicity of the PLA scaffold, which improves cell attachment and proliferation [20, 26].

Previously, the potential of scPLCL has been shown for vaginal reconstruction *in vitro* [16]. In this study, our aim was to further develop the scPLCL scaffold for vaginal reconstruction by embedding A2P into the scPLCL scaffold (scPLCL_A2P_). The effect of the scPLCL_A2P_ scaffold on the attachment, viability, proliferation, and collagen production of ECs and SCs in mono- and cocultures was evaluated. In particular, we evaluated whether A2P affects collagen production in SCs compared to plain scPLCL. To our knowledge, there are no previous studies evaluating the effect of A2P-releasing scaffolds for vaginal reconstruction.

## Materials and methods

### Scaffold manufacturing

The scaffolds were manufactured as described previously [16, 17]. Briefly, 8 wt.% A2P (Sigma-Aldrich Chemie Gmbh, Steinheim, Germany) was melt-mixed with PLCL 70 L/30CL (PLCL 7015, Corbion Purac BV, Gorinchem, Te Netherlands) in a twin-screw extrusion process. The extruded rods were foamed with scCO_2_ (Waters Operating Corporation, Milford, MA, USA) using high pressure and temperature. Thereafter, the foamed rods were cut into discs with a thickness of 3–3.5 mm, resulting in scPLCL_A2P_ porous scaffolds containing 4.7 ± 0.4 mg of A2P. Finally, the scaffolds were gamma-irradiated for sterility with a minimum dose of 25 kGy before the cell culture experiments. The porous scPLCL scaffolds were manufactured similarly, excluding melt mixing of A2P.

### MicroCT (μCT)

The porosity, pore sizes, and interconnectivity of the samples were analyzed with X-ray microtomography (μCT). The samples were imaged with a Zeiss Xradia MicroXCT-400 (Carl Zeiss X-ray Microscopy Inc., Pleasanton, CA, USA) device. A total of 1601 projections were acquired with one second of exposure time. The source voltage was 80 kV, and the current was 125 µA. The reconstruction was made with XMReconstructor software, resulting in a pixel size of 5.64 µm. Image processing and visualizations were performed with Avizo 2022.2 software (Thermo Fisher Scientific, Waltham, MA, USA). The pore size analysis was based on the BoneJ plugin in Fiji software [32]. The pore network and interconnectivity calculations were performed with an in-house MATLAB (The MathWorks Inc., Natick, MA, USA) program, which is described in more detail in [33]. The scPLCL_A2P_ scaffold particle segmentation, labeling and measurement were performed with Avizo software. Because of the imaging resolution, particles that were smaller than 500 µm^3^ in volume were excluded from the analysis.

### Cell isolation and seeding

Human ECs and SCs for this study were obtained from 3 different donors. Cell isolation was performed as described previously [16]. Briefly, the tissue samples were cut into small pieces and digested in a solution of collagenase and dispase. The suspension was filtered, centrifuged, and plated in a CellBind T75 flask (Sigma-Aldrich, St. Louis, MO, USA) with EpiLife™ medium (Invitrogen, Thermo Fisher Scientific, Waltham, MA, USA) supplemented with 1% EpiLife™ defined growth supplement (EDGS; Invitrogen), 0.1% CaCl2 (Invitrogen) and 0.35% antibiotics (100 U/ml penicillin and 0.1 mg/ml streptomycin [Lonza, BioWhittaker, Verviers, Belgium]). After primary culturing, the EC and SC lines were separated, and the cells were treated with TrypLE Select (Gibco, Thermo Fisher Scientific). The loosened cells were passaged to a T75 flask (Nunc, Thermo Fisher Scientific) in DMEM/F12 (basic medium, BM; Thermo Fisher Scientific) supplemented with 5% human serum (Biowest, Nuaille´, France), 1% GlutaMAX (Life Technologies, Thermo Fisher Scientific) and 1% antibiotics (100 U/ml penicillin and 0.1 mg/ml streptomycin; Lonza), resulting in the human vaginal SC line. The remaining cells, the human vaginal EC line, were further treated with TrypLE Select and passaged to T75 flasks with EpiLife™ medium. Both cell lines were passaged when confluent and frozen until experiments. ECs and SCs at passages 3–5 were used in the *in vitro* experiments.

Prior to cell seeding, the scPLCL_A2P_ scaffolds were prewetted in BM for 24 h at 37 °C and placed in the wells of 48-well plates (Nunc) for cell culture experiments. The experiments were performed either by culturing ECs or SCs as monocultures in separate scaffolds or coculturing the ECs and SCs on opposite sides of the same scaffold. In monocultures, 100,000 vaginal ECs or SCs in 15 μl of medium were plated on both sides of the scPLCL_A2P_ scaffold, and the cells were allowed to adhere for 2 h after 500 ml of EpiLife™ or BM was added, respectively. For cocultures, 100,000 SCs were plated on the scPLCL_A2P_ scaffold in 15 μl of medium and precultured for 5 days in BM. After 100,000 ECs were plated in 15 μl of medium on the other side of the scPLCL_A2P_ scaffold, the cocultures were cultured on EpiLife™ medium.

The cells were incubated in a humified atmosphere of 5% CO_2_ in air at 37 °C until analysis, and the specific medium was changed three times per week. The analyses were performed after 1, 7 or 14 d of cell culture. For cocultures, the time points followed the implantation of ECs; therefore, the corresponding culturing days for SCs in cocultures were 6, 12 or 19 days. The experiments and time points in mono- and/or cocultures are represented in the Table 1.

**Table 1:** Overview of the analyses, samples and timepoints

Analysis	Cell type	Time point
*Monoculture*		
SEM	EC/SC	1, 7 and 14 d
Live/dead	EC/SC	1, 7 and 14 d
CyQuant	EC/SC	1, 7 and 14 d
Sircol	EC/SC	14 d
qRT-PCR	EC/SC	14 d
Immunostaining		
Col I	SC	7 and 14 d
αSMA	SC	7 and 14 d
*Coculture*		
Live/dead	EC and SC	1, 7 and 14 d
Pancytokeratin-phalloidin	EC and SC	7 and 14 d

The SEM imaging, live/dead analysis, CyQuant proliferation assay, Sircol assay and qRT-PCR were done for both ECs and SCs in monocultures. The Col I and αSMA immunostaining were done for SCs. The outcome of EC and SC coculturing was evaluated with live/dead and pancytokeratin–phalloidin stainings

SEM = scanning electron microscopy, qRT–PCR = quantitative real-time polymerase chain reaction, Col I = collagen I, **α**SMA = actin α– smooth muscle, EC = human vaginal epithelial cell, SC = human vaginal stromal cell

### Scanning electron microscopy (SEM)

To evaluate the attachment and morphology of the ECs and SCs in monocultures, scanning electron microscopy (SEM) imaging was performed at d 1, d 7, and d 14 [16]. Briefly, the cells were fixed with 5% glutaraldehyde (Sigma-Aldrich) in 0.1 M phosphate buffer (pH 7.4, Sigma-Aldrich) at room temperature (RT) for 48 h. Then, the samples were dehydrated through a sequence of increasing concentrations (30, 50, 70, 80, 90. 95 and 100%) ethanol for 5 min and finally in a solution of 1:2 hexamethyldisilazane (HMDS, Sigma-Aldrich) and 100% ethanol (Altia Oyj, Helsinki, Finland) for 20 min following incubation in 2:1 HMDS and ethanol for 20 min. The samples were allowed to evaporate overnight in a fume, carbon sputtered and imaged with SEM (Zeiss ULTRAplus, Oberkochen, Germany).

### Viability staining and cell proliferation

The viability of ECs and SCs in both mono- and cocultures was studied at d 1, d 7, and d 14 time points with qualitative live/dead viability staining as described previously [13, 16]. The samples were incubated at 0.5 mM CalceinAM (green fluorescence; Molecular Probes) and 0.25 mM EthD-1 (green fluorescence; Molecular Probes) in DBPS (Sigma-Aldrich) for 45 min at RT and imaged with a fluorescence microscope (Olympus IX51S8F-2; camera DP71, Tokyo, Japan). The viable cells were stained green, and the dead cells were stained red. The background fluorescence caused by the scaffold without cells was used as a negative control.

The CyQUANT™ cell proliferation assay kit (Invitrogen) measuring the total DNA amount was used to evaluate the proliferation of ECs and SCs in monocultures at d 1, d 7, and d 14 as previously described[16]. Briefly, the cells were lysed with 0.1% Triton-X-100 buffer (Sigma-Aldrich) and stored at − 70 °C until analysis. After the freeze–thaw cycle, the working solution containing CyQUANT™ GR dye and cell lysis buffer was added, and the fluorescence at 480/520 nm was measured with a Victor 1420 Multilabel Counter microplate reader (Wallac, Turku, Finland).

### Total collagen content

The total soluble collagen content of ECs and SCs in monocultures was evaluated using the Sircol™ Soluble Collagen Assay (Biocolor, Carrickfergus, United Kingdom) measuring mammalian type I-V collagen at the d 14 time point, as described previously [18, 34]. Briefly, the samples were incubated in 0.5 M acetic acid (Merck, Darmstadt, Germany) with 0.1 mg/ml pepsin (Sigma-Aldrich) for 4 h at + 4 °C to extract the acid-soluble collagen from samples. Sircol Dye reagent (Biocolor) was added to liquid samples and incubated with gentle shaking for 30 min at RT. The samples were centrifuged for 10 min at 12 000 rpm, and collagen pellets were washed with ice-cold Acid-Salt Wash Reagent (Biocolor). The samples were centrifuged again, and the dyed collagen was further dissolved in 0.5 M sodium hydroxide solution (Biocolor), and the intensity of the red dye was measured with a Victor 1420 microplate reader (Wallac) at 540 nm.

### Quantitative real-time polymerase chain reaction (qRT–PCR)

The quantitative relative expression of the genes cytokeratin (CK) 7 and CK19 for ECs and alpha smooth muscle actin (αSMA), elastin, collagen I (Col I) and collagen III (Col III) for SCs was studied using real-time reverse transcription-polymerase chain reaction (qRT–PCR). For qRT–PCR analysis, ECs and SCs were monocultured on scPLCL and scPLCL_A2P_ for 14 days, and polystyrene cell culture plastic (PS) without A2P served as a control as previously described [16, 34]. Briefly, the cells were lysed, and total RNA was isolated with Nucleospin kit reagent (Macherey–Nagel GmbH & Co. KG, Düren, Germany). Next, the mRNA was reverse transcribed to cDNA with a high-capacity cDNA Reverse Transcriptase Kit (Thermo Fisher Scientific). The qRT–PCR mixture contained cDNA, forward and reverse primers (Table 2, OligomerOy, Helsinki, Finland), and SYBR Green PCR Master Mix (Applied Biosystems, Foster City, CA, USA).

**Table 2:** The qRT–PCR primer sequences used in this study

Name	Primer	5′-sequence-3′	Product size (bp)	Accession number
CK7	Forward	CATCGAGATCGCCACCTACC	80	NM_005556.3
	Reverse	TATTCACGGCTCCCACTCCA		
CK8	Forward	CCATGCCTCCAGCTACAAAAC	68	M34225.1
	Reverse	AGCTGAGGTTTTATTTTGGACC		
CK19	Forward	ACTACACGACCATCCAGGAC	80	NM_002276.4
	Reverse	GTCGATCTGCAGGACAATCC		
αSMA	Forward	GAC AAT GGC TCT GGG CTC TGT AA	194	NM_001613.4
	Reverse	ATG CCA TGT TCT ATC GGG TAC TT		
Elastin	Forward	GGT GCG GTG GTT CCT CAG CCT GG	613	NM_000501.4
	Reverse	GGG CCT TGA GAT ACC CCA GTG		
Col I	Forward	CCA GAA GAA CTG GTA CAT CAG CAA	94	NM_000088.3
	Reverse	CGC CAT ACT CGA ACT GGA ATC		
Col III	Forward	CAG CGG TTC TCC AGG CAA GG	179	NM_000090
	Reverse	CTC CAG TGA TCC CAG CAA TCCC		
RPLP0	Forward	AAT CTC CAG GGG CAC CATT	70	NM_001002
	Reverse	CGC TGG CTC CCA CTT TGT		

The reaction was conducted with an AbiPrism 7000 Sequence Detection System (Applied Biosystems), and the initial enzyme activation was performed at 95 °C for 10 min, followed by 45 cycles of denaturation at 95 °C for 15 s and annealing and extension at 60 °C for 60 s. The gene expression levels of CK7, CK8, CK19, αSMA, elastin, Col I and Col III were normalized to the expression of the housekeeping gene large ribosomal protein P0 (RPLP0), and the relative expression was calculated using a previously described mathematical model [35]. Furthermore, the Col I/Col III ratio was calculated by using average qRT–PCR cycle threshold (Ct) values for Col I and Col III mRNA.

### Immunostaining

The staining of Col I (anti-collagen I antibody, 1:2000, Abcam, Cambridge, UK) and αSMA (anti-actin α-smooth muscle, 1:400, Sigma) was evaluated after 7 and 14 d in SC monocultures on scPLCL and scPLCL_A2P_. Additionally, in cocultures of ECs and SCs on scPLCL_A2P_, the staining of pancytokeratin (AE1/AE3, 1:250, Cytokeratin Pan Ab, Thermo Fisher Scientific) and actin cytoskeleton organization (phalloidin-tetramethylrhodamine B isothiocyanate, 1:500, Sigma-Aldrich) was evaluated after 7 and 14 d of cell culturing.

The samples were fixed with 0.2% Triton X-100 (Sigma-Aldrich) in 4% PFA (Sigma-Aldrich) and incubated overnight in the abovementioned primary antibody dilutions. The following day, the SC monocultures were incubated in secondary antibody dilutions (1:400, goat anti-mouse IgG1 or 1:300, goat anti-mouse IgG (H + L), Alexa-fluor 488, green fluorescence, Invitrogen). The EC and SC cocultures were incubated in a mixture of secondary antibody (1:400, goat anti-mouse IgG (H + L), Alexa-fluor 488, green fluorescence, Invitrogen) and phalloidin. Finally, the cell nuclei were stained with DAPI (1:200, blue fluorescence, Sigma-Aldrich), and the samples were imaged with a fluorescence microscope (Olympus).

### Statistical analysis

Statistical analyses were performed with SPSS v 23 (IBM SPSS Statistics for Windows, Armonk, NY, USA). The CyQUANT™ cell proliferation assay was repeated with cells from three different donors using three parallel cell samples and three technical replicates (n = 27). The change in cell amount in relation to culturing time points was analyzed. The total collagen content measured with the Sircol™ collagen assay was repeated using cells from three different donors with three parallel cell samples and two technical replicates (n = 18). qRT–PCR was repeated with cells from three different donors using two parallel samples; however, some expression values were required to be removed from the analyses (n = 5–6). The differences in gene expression between the materials were evaluated. The data were nonnormally distributed; therefore, nonparametric Kruskal–Wallis (CyQuant and qRT–PCR) or Mann–Whitney (Sircol) tests were used to analyze the data in both analyses and are reported as medians and quartiles (*p* < 0.05 considered significant).

## Results

### ***The*** μ***CT characterization of the scPLCL***_***A2P***_*** scaffold***

**Fig. 1:**
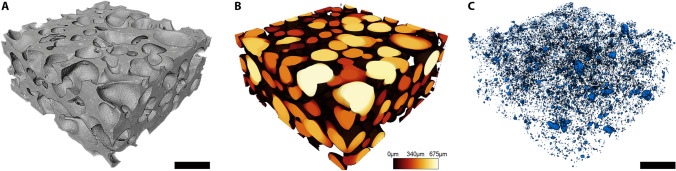
Representative μCT images of scPLCL_A2P_ scaffolds. The images illustrate the structure of the porous scPLCL_A2P_ scaffold (**A**), the size and distribution of pores in the scaffold (**B**) and the distribution of the A2P particles (**C**)

μCT imaging was used to characterize the scPLCL_A2P_ scaffolds (n = 3). The average porosity of the scPLAL_A2P_ scaffolds was 59.6 ± 3.3%, and the average pore size was 346 ± 123 μm. The pores were evenly distributed in the scaffold, as shown in Fig. 1.

**Fig. 2:**
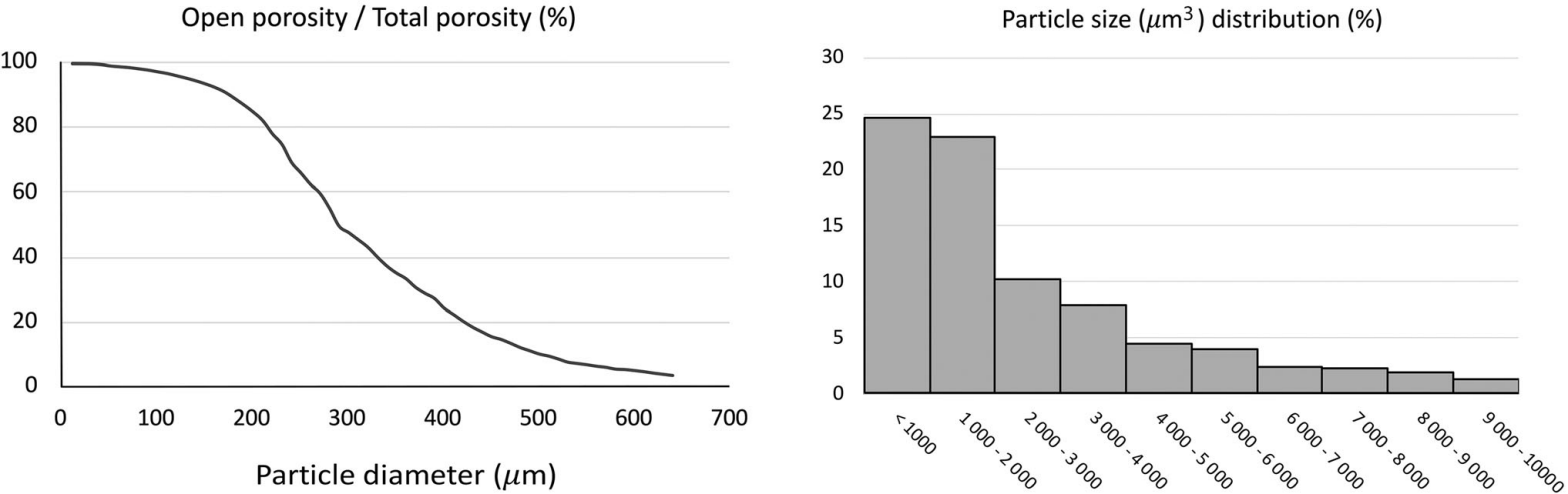
The interconnectivity percentage (left image) of the pores is illustrated as a proportion of open porosity in relation to the total porosity. The interconnectivity percentage is represented as a function of particle diameter to enable penetration through the pores outside the scaffold. The right image represents the percent distribution of A2P particle size in the scPLCL_A2P_ scaffold

Furthermore, the interconnectivity of the pores was high, and a spherical object with a diameter of 100 μm reached 97% of the scaffold’s total pore space from outside the scaffold (Fig. 2). The particle size distribution was also analyzed: 70.2% of the A2P particles were 5000 µm^3^ or smaller, and the particles were evenly distributed throughout the scaffold (Figs. 1 and 2).

### ***The ECs and SCs retained their morphology in scPLCL***_***A2P***_

**Fig. 3:**
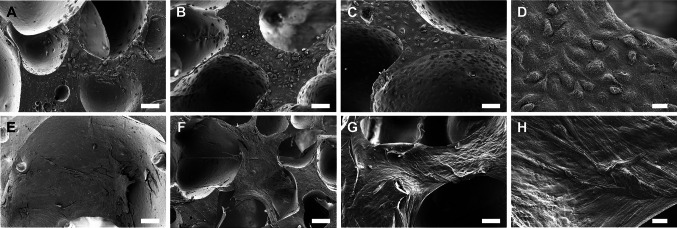
SEM images of ECs (**A**–**D**) and SCs (**E**–**H**) on the scPLCL_A2P_ scaffold after 1 d (**A**, **E**), 7 d (**B**, **F**) and 14 d (**C**, **D**, **G**, **H**) of cell culture. Both ECs and SCs attached and spread on the scaffold surface and retained their morphology during the 14-d culture period. Scale bar 100 μm (**A**–**C**, **E**–**G**). Figures **D** and **H** (scale bar 20 μm) show higher magnification of the EC and SC morphology at the d 14 time point

The EC and SC attachment, spreading and morphology in monocultures were studied after 1 d, 7 d and 14 d of cell culture with SEM imaging (Fig. 3).

The ECs were already attached and spread on the surface of the scaffold at d 1 (Fig. 3A), and the cells were flattened and cuboidal. After 7 d (Fig. 3B) and 14 d (Fig. 3C, D), the ECs were spread and attached to the whole scPLCL_A2P_ scaffold, and ECs obtained their characteristic small, oval, roundish or polygonal morphology. Furthermore, the ECs seemed to retain their epithelial morphology during the 14-d culture period.

In addition, the SCs were attached and flattened on the surface of the scPLCL_A2P_ scaffold after 1 d (Fig. 3E) of cell culture, and the number of spherical SCs was minor. At the d 7 time point (Fig. 3F), the SCs were spread on the surface of the scaffold, and no cell clusters were detected. The cells also formed cell bridges over the scaffold pores. After 14 d of cell culture (Fig. 3G, H), a flat SC layer covered the surface of the scPLCL_A2P_ scaffold, and no cell clumps were detected. Furthermore, the SCs formed cell bridges over the scaffold pores and formed an even cell layer over the pores. During the whole 14-d culture period, the SCs retained their morphology, and the cells were flat and spindle-shaped or multipolar. The SCs on the edge of the pore were more spindle shaped and grew parallel to each other, whereas on the flat surface or in cell layers covering the pore, the cells seemed more multipolar, formed cell layers and grew in different directions compared to adjacent cells.

### ***scPLCL***_***A2P***_*** supports the viability and proliferation of ECs and SCs***

**Fig. 4:**
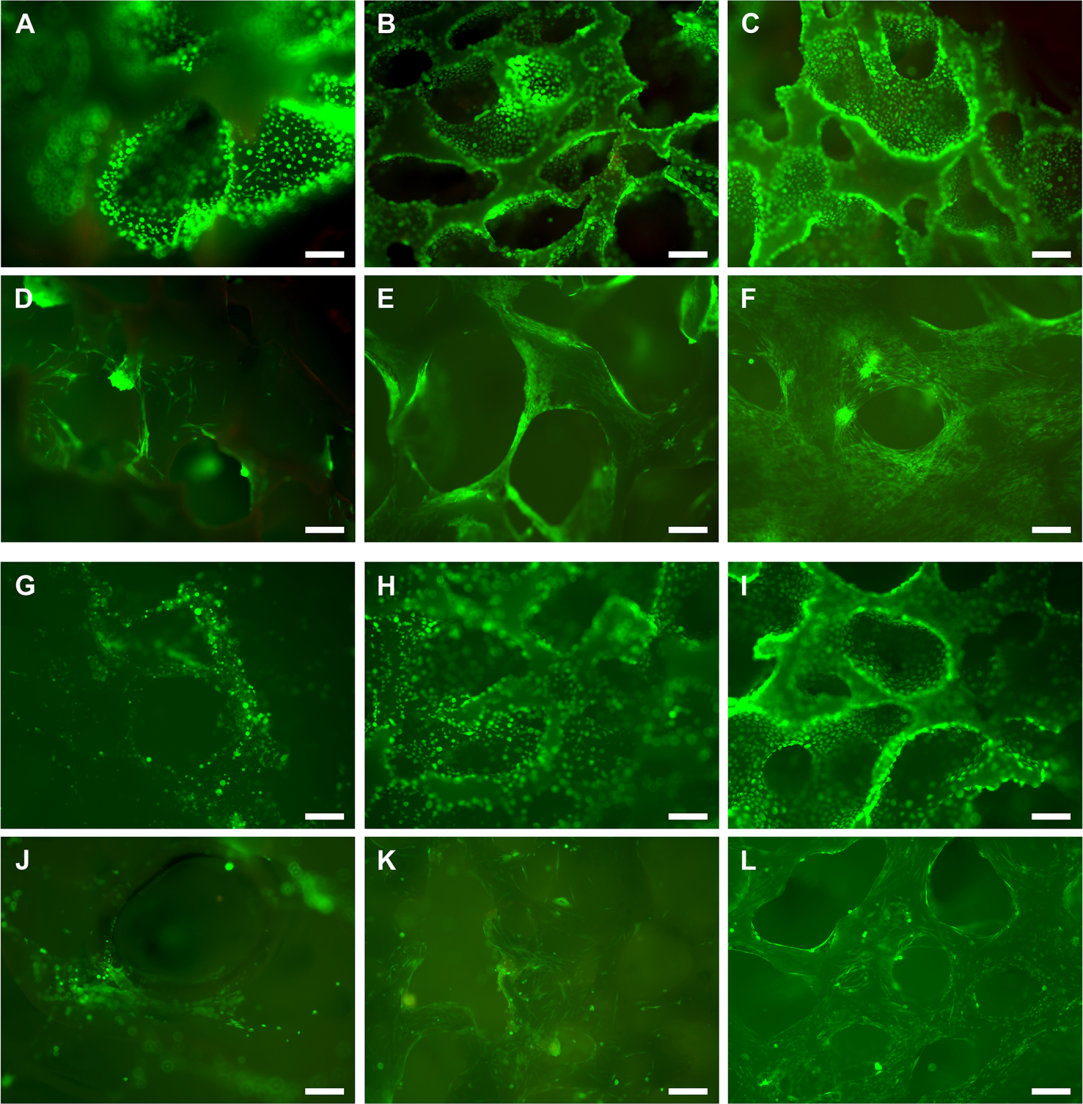
The viability of ECs (**A**–**C**, **G**–**I**) and SCs (**D**–**F**, **J**–**L**) on scPLCL_A2P_ in monocultures (**A**–**F**) and cocultures (**G**–**L**). The ECs and SCs were viable (green fluorescence) at 1 d (**A**, **D**, **G**, **J**), 7 d (**B**, **E**, **H**, **K**) and 14 d (**C**, **F**, **I**, **L**), and hardly any dead cells (red fluorescence) were detected. In cocultures, the SCs also remained viable under suboptimal cell culture conditions in epithelial medium (**J**–**L**). Scale bar, 250 μm

The viability of ECs and SCs in mono- and cocultures was evaluated at d 1, d 7 and d 14 time points using live/dead viability staining (Fig. 4). In monocultures, both ECs (Fig. 4A–C) and SCs (Fig. 4D–F) maintained their viability, and hardly any dead cells were visible throughout the whole culture period. The viability staining also illustrates that both cell types evenly covered the scPLCL_A2P_ scaffold. Further, the number of SC clusters was minimal. In cocultures, both ECs (Fig. 4G–I) and SCs (Fig. 4J–L) remained viable during the 14-d coculturing period, and the number of dead cells was low. However, based on the live/dead images, the amount of SCs seemed lower after 14 d of coculture compared to that in monoculture.

**Fig. 5:**
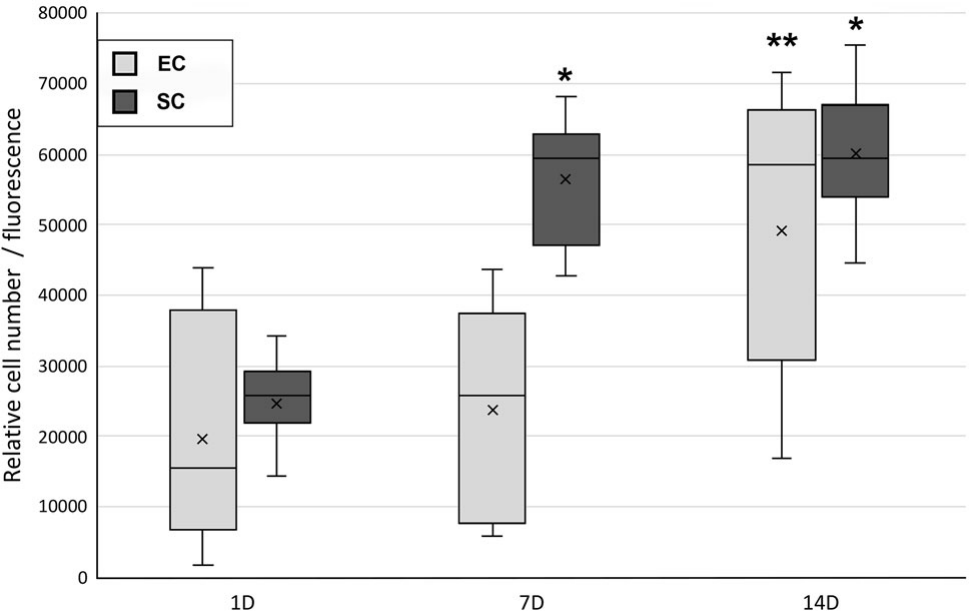
The proliferation of ECs and SCs on scPLCL_A2P_ was evaluated with a CyQUANT™ cell proliferation assay (n = 27). The number of SCs was significantly higher on d 7 and d 14 than on d 1 (*p* < 0.001, marked with *). Furthermore, the relative number of ECs was significantly increased after 14 d compared to 1 or 7 d (*p* < 0.001, marked with **)

The proliferation of EC and SC in monocultures was quantitatively evaluated by measuring the DNA amount in monoculture samples after 1, 7 and 14 d of cell culture (Fig. 5, n = 27). According to quantitative assessment, the ECs proliferated during the 14-d culture period, and at d 14, the cell number was significantly higher than that at the d 1 or d 7 time points (*p* < 0.001). In SC monocultures, the cell number significantly increased from d 1 to d 7 or d 14 (*p* < 0.001). However, no significant increase was detected in SC monocultures between d 7 and d 14.

### ***scPLCL***_***A2P***_*** increases the collagen production of SCs compared to scPLCL***

**Fig. 6:**
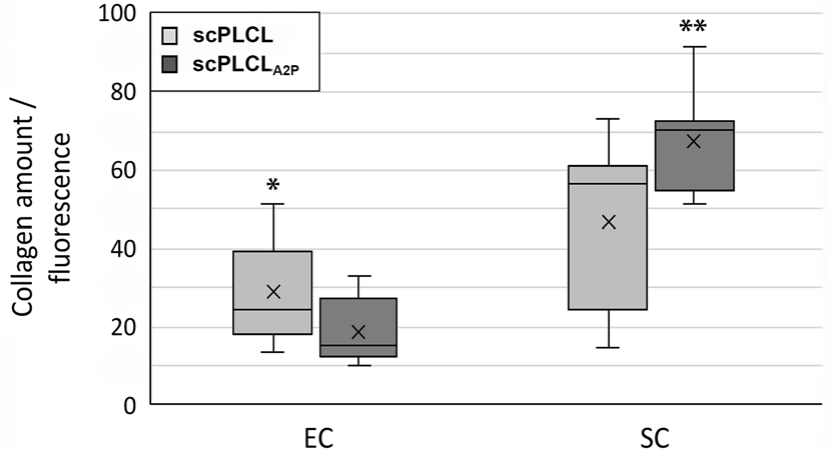
The amount of collagen secreted by ECs and SCs on scPLCL or scPLCL_A2P_ after 14 d of cell culture (n = 18). Interestingly, the ECs on scPLCL secreted more collagen than the ECs on scPLCL_A2P_ (*p* = 0.005, marked with *). The SC collagen secretion on scPLCL_A2P_ was significantly higher than on scPLCL (*p* = 0.005, marked with **)

In EC and SC monocultures, the quantitative Sircol™ assay was used to compare the collagen production of cells on the scPLCL_A2P_ scaffold and plain scPLCL scaffold. The amount of soluble collagen (types I–V) was evaluated after 14 d of cell culture in EC and SC (Fig. 6, n = 18). According to the Sircol assay, embedding A2P on the scPLCL scaffold increased SC collagen production compared to plain scPLCL (*p* = 0.005). Interestingly, the effect of A2P is opposite that of ECs, and the amount of collagen produced by ECs decreases on scPLCL_A2P_ scaffolds compared to plain scPLCL scaffolds (*p* = 0.005).

### A2P increases the expression of Col I and III genes in SCs

**Fig. 7:**
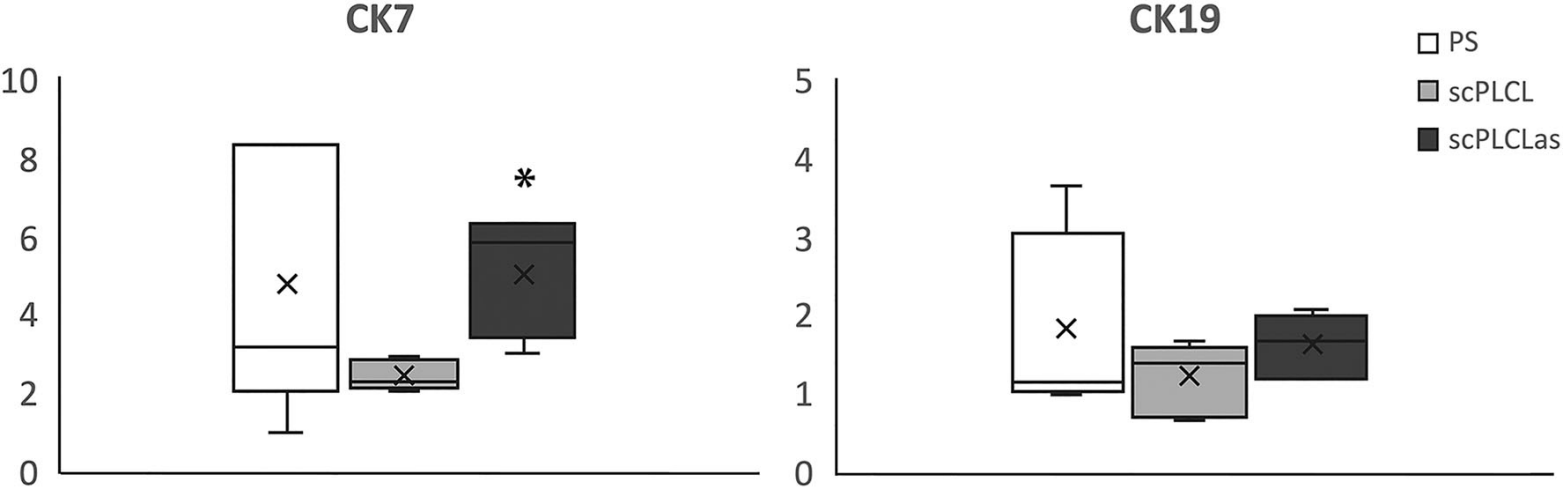
The ECs’ relative expression of epithelial markers CK7 and CK19 on scPLCL and scPLCL_A2P_ (PS serving as a control) was evaluated after 14 d of cell culturing (n = 5–6). The CK7 expression of ECs was higher for scPLCL_A2P_ than for scPLCL (*p* = 0.02, marked with *)

**Fig. 8:**
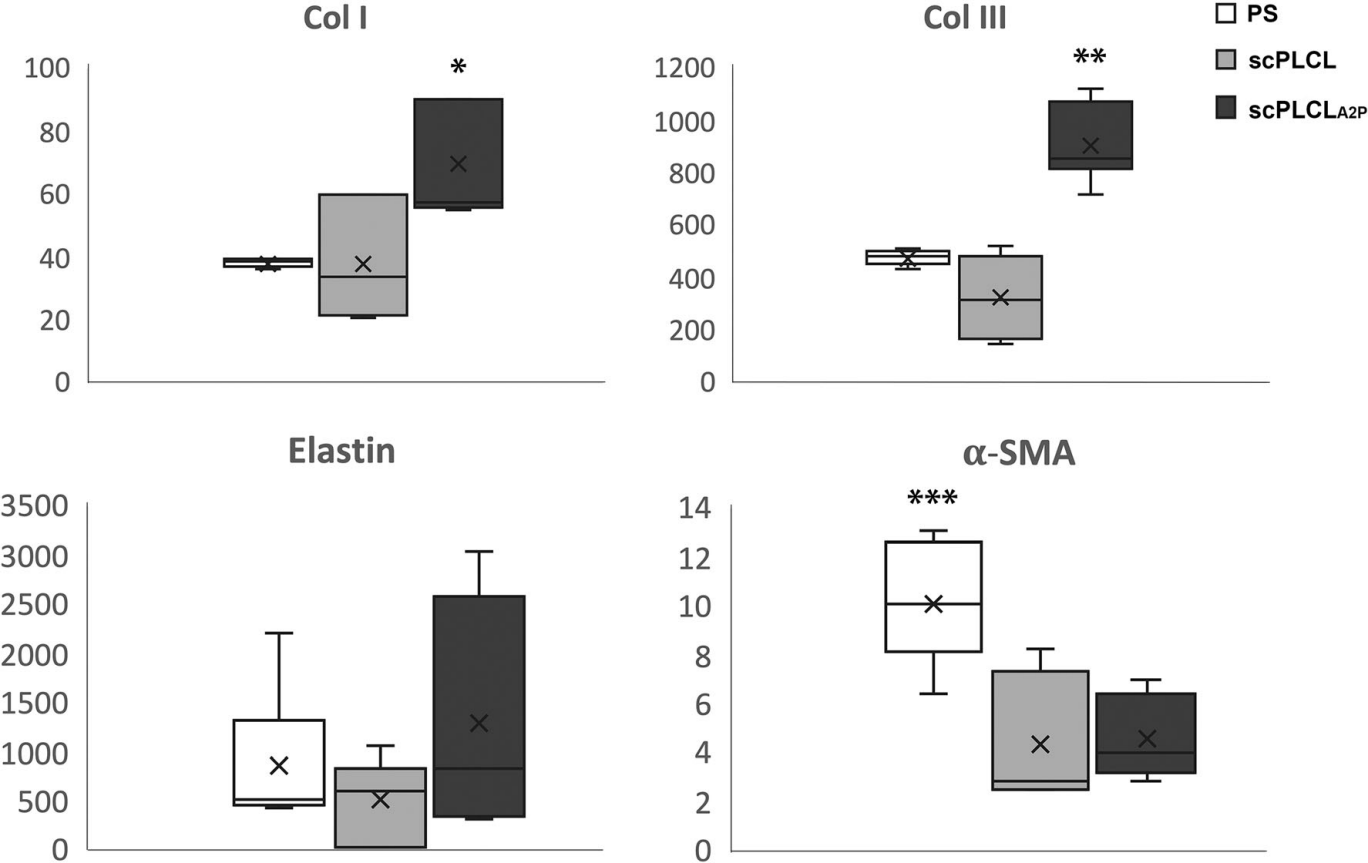
The relative expression of the SC markers Col I, Col III, elastin and αSMA on scPLCL and scPLCL_A2P_ (PS served as a control) was evaluated after 14 d of cell culture (n = 5–6). The SCs on scPLCL_A2P_ expressed significantly more collagen I than those on scPLCL (*p* = 0.017, marked with *). Collagen III expression was higher on scPLCL_A2P_ than on the other materials (*p* = 0.001 for scPLCL and 0.017 for PS, marked with **). However, there were no significant differences in elastin expression, and SCs on PS expressed αSMA more than scPLCL (*p* = 0.004, marked with ***) and scPLCL_A2P_ (*p* = 0.013, marked with ***)

The expression of epithelial (CK7 and CK19) and stromal (elastin, Col I, Col III and αSMA) marker genes in EC and SC monocultures, respectively, were compared on scPLCL_A2P_, plain scPLCL and PS (Figs. 7 and 8, n = 5–6) after 14 d of cell culture. The expression of CK7 in ECs was significantly higher on scPLCL_A2P_ than on scPLCL (*p* = 0.02), but there was no difference in CK19 expression.

The expression levels of Col I (*p* = 0.017) and Col III (*p* = 0.001) in SCs were higher on scPLCL_A2P_ than on scPLCL. However, the average Col I/Col III mRNA ratios were similar between the studied samples: 0.96 for the PS control, 0.94 for the scPLCL scaffold and 0.98 for the scPLCL_A2P_ scaffold (n = 3). Due to the small sample size, no statistical analysis was performed for the Col I/Col III ratio. Furthermore, SC αSMA expression was higher on PS than on both scPLCL (*p* = 0.004) and scPLCL_A2P_ (*p* = 0.013). There were no significant differences in elastin expression between the samples.

### ***The staining of Col I and*** α***SMA in SC monocultures and pancytokeratin expression in cocultures***

**Fig. 9:**
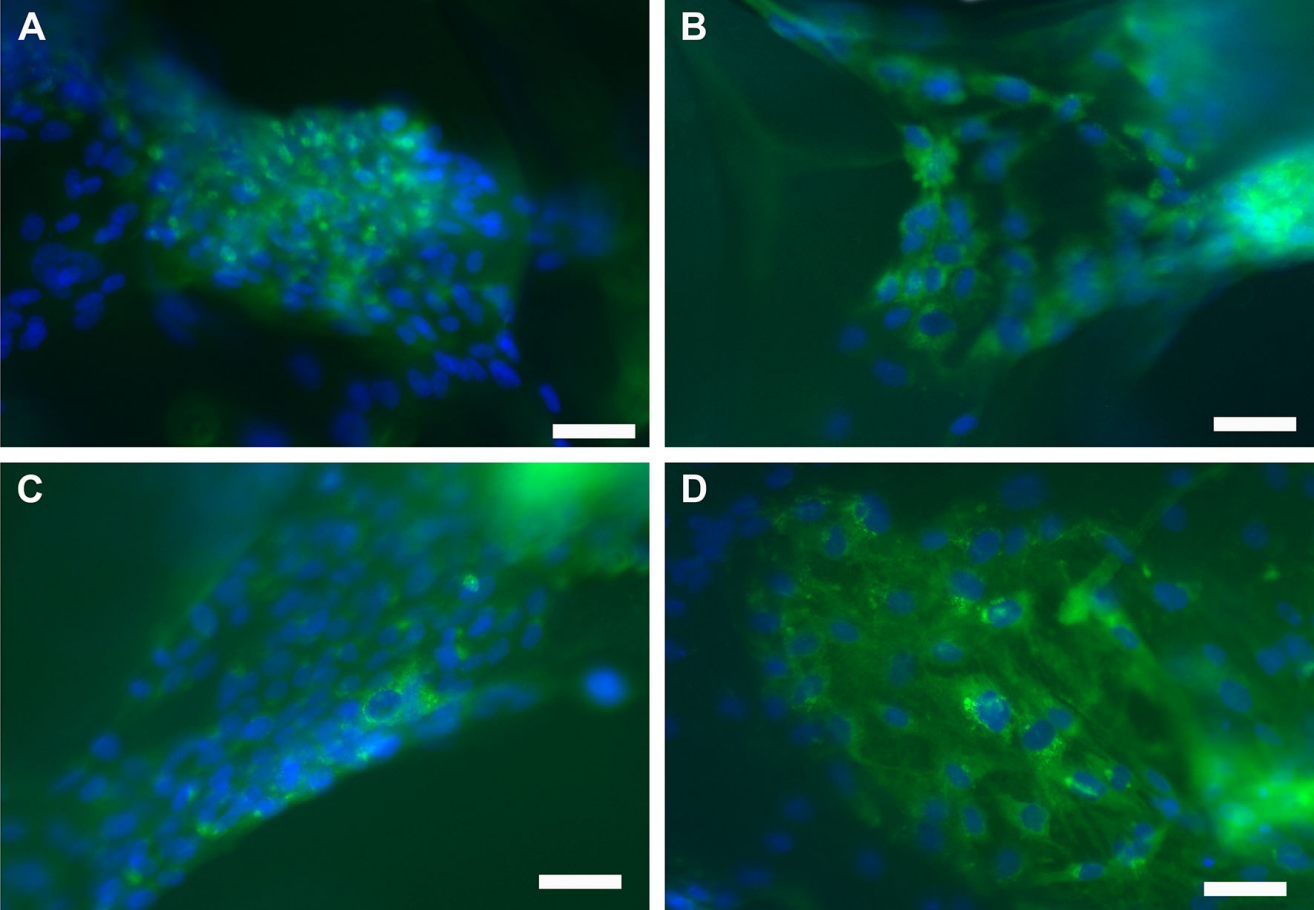
Representative images of SC Col I staining on scPLCL (**A**, **B**) and scPLCL_A2P_ (**C**, **D**) after 7 d (**A**, **C**) and 14 d (**B**, **D**) of cell culture. There were no substantial differences in Col 1 staining between the materials. Scale bar 50 μm

**Fig. 10:**
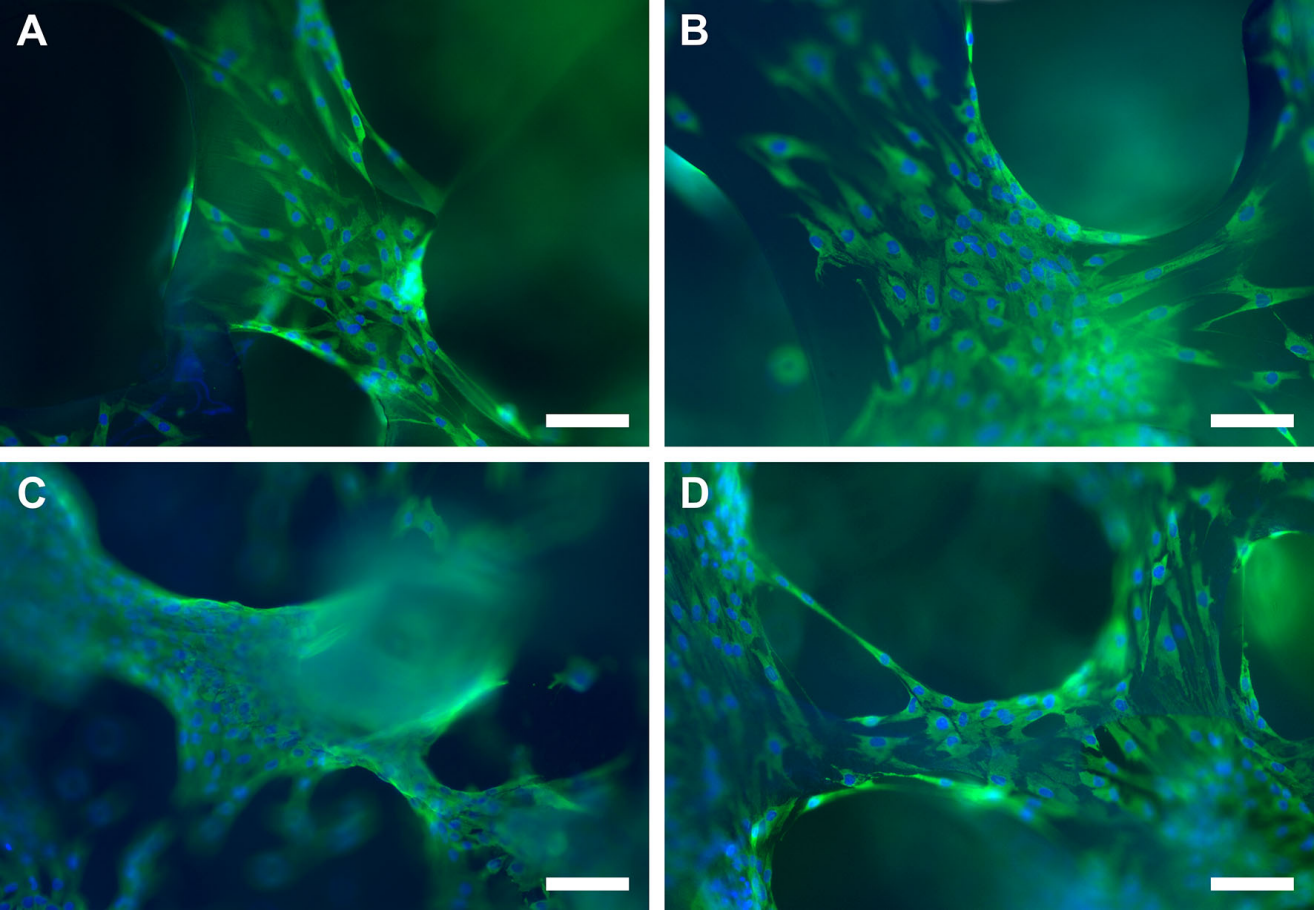
αSMA staining of SCs on scPLCL (**A**, **B**) and scPLCL_A2P_ (**C**, **D**) after 7 d (**A**, **C**) and 14 d (**B**, **D**) of cell culture. There were no substantial differences in the intensity of αSMA staining between scPLCL and scPLCL_A2P_. Scale bar 50 μm

The staining of Col I (Fig. 9) and αSMA (Fig. 10) on scPLCL and scPLCL_A2P_ was evaluated in SC monocultures after 7 d and 14 d of cell culture. At d 7 or d 14, the SCs were stained with Col I, and no substantial qualitative difference in the staining intensity was detected between scPLCL_A2P_ (Fig. 9A, B) and plain scPLCL (Fig. 9C, D). Furthermore, there was no substantial difference in the level of Col I staining intensity between the d 7 and d 14 time points for either scPLCL or scPLCL_A2P_. The αSMA staining of SCs was intensive on both scPLCL and scPLCL_A2P_ scaffolds, and no distinctive differences were detected. Additionally, the αSMA staining intensity remained constant between d 7 (Fig. 10A, C) and d 14 (Fig. 10B, D).

**Fig. 11:**
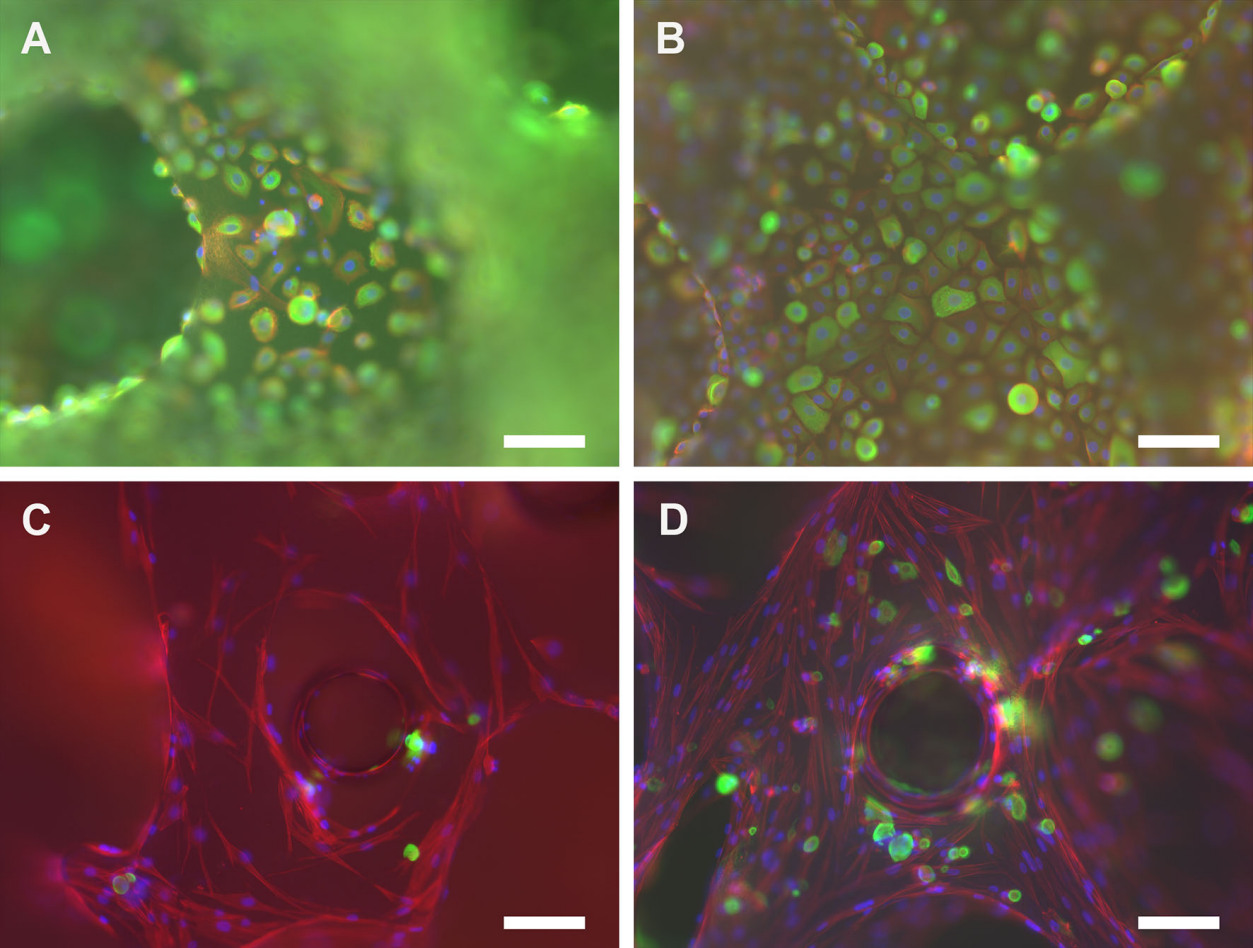
Pancytokeratin staining of ECs (**A**, **B**) and actin cytoskeleton organization of ECs (**A**, **B**) and SCs (**C**, **D**) were evaluated after 7 d (**A**, **C**) and 14 d (**B**, **D**) of coculture on scPLCL_A2P._ The staining of pancytokeratin remained constant during the assessment period. Based on actin cytoskeleton staining, SCs maintain their actin organization even under suboptimal cell culture conditions in epithelial medium. Furthermore, some ECs (green staining) were also evident on the SC side of the scaffolds

In EC and SC cocultures on scPLCL_A2P_, AE1/AE3 pancytokeratin and actin cytoskeleton staining was used to evaluate the maintenance of the epithelial phenotype and cytoskeleton organization at d 7 (Fig. 11A, C) and d 14 (Fig. 11B, D), respectively. The pancytokeratin staining of ECs was intense at both time points and remained constant between d 7 and d 14. The actin filaments on ECs are aligned on the cell edges, whereas on SCs, the actin filaments are spread into the SC cytoplasm and aligned in a parallel manner. Moreover, no changes in actin filament organization were detected between d 7 and d 14.

## Discussion

The surgical reconstruction of vaginal defects with existing methods is highly challenging and susceptible to complications such as graft shrinkage and stenosis. Moreover, the need for vaginal reconstruction is emerging due to the increasing amount of transgender surgery. Thus, the development of new and safe treatment methods for the reconstruction of large vaginal defects and deformities is highly important. To conquer the problems related to acellular nonvaginal tissue grafts, we aim to develop cell-seeded tissue engineering-based scaffolds for vaginal reconstruction [5, 6]. Here, we studied the A2P-releasing scPLCL scaffold seeded with vaginal ECs and SCs in mono- and cocultures. A2P was embedded into scPLCL to increase the collagen production of SCs, facilitating stromal layer formation in tissue-engineered neovaginas, since effective stromal regeneration is known to prevent graft shrinkage and fibrosis [36, 37]. To our knowledge, A2P-releasing scaffolds have not been previously studied for vaginal tissue engineering.

In addition to biodegradable polymer materials, the scaffold manufacturing method is essential in applications aimed at clinical solutions. Supercritical carbon dioxide (scCO_2_) foamed scaffolds have been previously studied for vaginal, urothelial and cartilage applications with encouraging results [16, 19, 34]. The scCO_2_ foaming allows the control of pore size without any toxic solvents using controlled temperature and pressure, and based on the μCT results, the scPLCL_A2P_ scaffolds manufactured for this study had an average porosity of 59.6 ± 3.3% and pore size of 346 ± 123 μm. The average porosity of A2P-embedded scaffolds was slightly lower than that in previous study with plain scPLCL scaffolds [16, 34]. The μCT imaging illustrated that the open porosity/total porosity was similar on A2P-containing scaffolds, where balls with a diameter of 100 μm can reach 97% of the scaffold pores, compared to plain scPLCL scaffolds, in which the corresponding interconnectivity was 98% [16]. Thus, the interconnectivity of the pores did not decline due to the embedded A2P, as described previously [34]. This was also supported by the finding that in coculture, the ECs were remotely detected on the SC side of the scaffold in pancytokeratin–phalloidin immunostaining. Additionally, the μCT analysis showed that the A2P particles presented a small particle size and an even distribution throughout the scaffolds, representing homogeneity of the melt mixing.

Targeting vaginal epithelial regeneration, the morphology, viability, and proliferation of ECs on scPLCL_A2P_ were evaluated. According to the SEM and live/dead imaging, the ECs were attached to the surface of the scPLCL_A2P_ scaffold after 1 d of cell culturing, being cuboidal or roundish in morphology typical of epithelial cells [13, 16, 34]. Moreover, the ECs retained their viability and morphology and spread evenly on the scaffold surface during the 14-d culture period in both mono- and cocultures on the A2P-releasing scPLCL scaffold and compared to the previous results with the plain scPLCL scaffold, no distinctive differences were detected [16]. Furthermore, qualitative evaluation with live/dead and SEM imaging in monocultures revealed that the number of ECs increased during the assessment period, which was confirmed with the quantitative CyQUANT® cell proliferation assay, indicating the concordance of different analyses and the compatibility of scPLCL_A2P_ for ECs. Previously, the epithelial cell compatibility of the scPLCL_A2P_ scaffold was evaluated only with human urothelial cells (hUC) [34]. Based on live/dead staining, the coculturing of ECs with SCs on scPLCL_A2P_ did not deteriorate EC viability or qualitatively assessed proliferation compared to monocultures, which was previously demonstrated for hUCs cocultured with human adipose stromal cells (hASCs) [34].

CK7 and CK19 are markers expressed widely in different epithelial cell layers of stratified epithelium [38]. Therefore, the maintenance of the EC epithelial phenotype on scPLCL_A2P_ was compared to that on plain scPLCL in monocultures with qRT–PCR expression of CK7 and CK19. The CK7 expression of ECs in scPLCL_A2P_ was significantly higher than that in plain scPLCL, which might indicate the maturation of the ECs as an effect of the released A2P. Signs of epithelial maturation were also detected in hUCs cultured on the A2P-releasing scPLCL scaffold [34]. However, no difference in CK19 expression was detected in ECs on the scaffolds. In addition to monocultures, EC phenotype maintenance was evaluated in cocultures with SCs using pancytokeratin immunostaining. As previously detected with hUCs, the ECs stained concordantly with pancytokeratin [34]. No distinct difference was detected in the intensity of staining between the d 7 and 14 time points, indicating that the scPLCC_A2P_ scaffold supports the maintenance of the EC epithelial phenotype in cocultures.

In SC monocultures, the SCs were flat and spread on the surface of the scPLCL_A2P_ scaffold after 1 d of cell culturing, and based on SEM analysis, they retained their morphology throughout the 14-d assessment period. After 14 days, the SCs covered the surface of the scaffolds and formed an even cell cover with parallel aligned SCs on the scPLCL_A2P_ scaffold. Additionally, based on the SC phalloidin staining in cocultures with ECs, the SCs were aligned by adapting the organization of the SC cytoskeleton, and no changes were detected between d 7 and 14 (Fig. 11). In previous study, with a plain scPLCL scaffold, the SCs formed cell clusters in both monocultures and cocultures [16]. In cocultures, this could be partially explained by the nonoptimal EpiLife™ medium used for cell culture in the coculture experiment. However, in the present study, with A2P-releasing scPLCL scaffolds, the SCs seemed to adhere to the material surface evenly, and only a few cell clusters were detected according to live/dead staining or SEM imaging, indicating that A2P facilitates cell adherence and spreading on the material surface [39]. In addition to monoculture, this tendency was also detected in cocultures, where SCs were cultured with ECs in nonoptimal cell culture medium conditions in EpiLife™ medium. The absence of cell clusters in cocultures further indicates the positive effect of A2P on SCs. A similar favorable effect of the A2P release scaffold was previously detected with hASCs [34]. The number of SCs increased during the 14-d cell culture period, which is concordant with previous results illustrating increased proliferation of stromal cells as an effect on AAD [23, 26, 34]. In particular, SCs proliferated rapidly within the first 7 d. As previously determined, the A2P-embedded scPLCL scaffolds release the A2P fastest within the first week; approximately 60% of the A2P was released during the first week of hydrolysis, reaching 70% release in two weeks, after which the release was markedly decelerated [17]. Based on these previous release results and our cell culture results, the A2P appears advantageous for cell proliferation, especially during the critical first week of cell culture.

AA has an essential role in collagen synthesis, and it has been shown to increase collagen production in cells *in vitro*. Mammalian cells are not able to synthetize AA, and therefore must be exogenously supplemented [26, 24] Embedding AA or its derivatives into biomaterial scaffolds provides new and interesting insight into the further development of biomaterial scaffolds for tissue engineering requiring an enhanced yield of collagen, which is one of the main fibrillar components in the vaginal wall extracellular matrix (ECM) [36]. In this study, we showed significantly higher collagen production of SCs on scPLCL_A2P_ scaffolds compared to plain scPLCL. The effect of the A2P-releasing scaffold on collagen yield was also previously detected with human dermal fibroblasts and hASCs [26, 34, 40]. Furthermore, the qRT–PCR results illustrated higher Col I and Col III gene expression in SCs cultured on scPLCL_A2P_ than in those cultured on plain scPLCL. Based on immunostaining with Col I, the SCs evidently stained with Col I on scPLCL_A2P,_ but based on the visual qualitative analysis, no substantial difference was detected compared to scPLCL (Fig. 9). However, the different analyses in this study concordantly demonstrated that the A2P-releasing scPLCL scaffold increased SC collagen production compared to plain scPLCL, as described in previous studies [26, 34, 40].

Additionally, there was no substantial difference in the Col I/Col III ratio between scaffolds and the control PS. Interestingly, in previous study with hASCs, A2P seemed to increase Col III production but not Col I production, indicating that the effect of A2P on collagen production might be stromal cell type specific [34]. The increase in Col III production increases the elasticity and laxity of the vaginal ECM, yet the increased production is also associated with scar tissue formation [36]. Here, the expression of both Col I and Col III were increased and no overexpression of Col III relative to Col I was detected, which might indicate the steady increase in both collagen types as an effect to the A2P, which could be favorable to constrain the formation of fibrosis. However, the optimal ratio is not yet known. Hung et al. showed that human vaginal fibroblasts had higher cell proliferation with a Col I/Col III ratio > 1 [41]. In epithelial cells, A2P has the opposite effect, and lower collagen production was detected with scPLCL_A2P_ scaffolds than with plain scPLCL scaffolds; this effect was previously also detected with urothelial cells [34]. However, epithelial cells are not intended to produce high amounts of collagen as stromal cells [36, 41].

The fibroblast–myofibroblast transition is a key factor in vaginal ECM remodeling that also affects healing after surgery [36]. Therefore, we analyzed the αSMA staining intensity with immunostaining and expression with qRT–PCR to detect the maintenance of the myofibroblast potential of the cultured SCs. The results showed that the SCs expressed αSMA intensively on scPLCL_A2P,_ and the intensity of staining remained constant between 7 and 14 d of culture, indicating the stability of the SC myofibroblast phenotype in both scaffolds. Interestingly, no significant difference in αSMA levels in SCs was detected between the scPLCL and scPLCL_A2P_ scaffolds either with immunostaining or qRT–PCR. In a previous study with hASCs, an increase in αSMA gene expression was detected in A2P-containing scaffolds, which might be due to the myoblastic differentiation of hASCs [42, 43]. In addition to collagen, elastin is the other major component in vaginal wall ECM comprising elasticity, and the increase in SC elastin expression would be beneficial for the elasticity of the neovagina [36]. However, we did not detect any significant differences between the scaffolds, even though the expression of elastin in scPLCL_A2P_ appeared slightly higher. The absence of a significant difference could be partially due to the small sample size and large deviation between cell lines, which is typical of primary cells.

In conclusion, on scPLCL_A2P_ scaffolds, both ECs and SCs maintained their morphology, viability, and phenotype during the 14-d assessment period in mono- and coculture conditions. Furthermore, both ECs and SCs increased their cell number *in vitro*. These results further illustrate the good biocompatibility of A2P-releasing scPLCL scaffolds. We demonstrated that the embedded A2P scaffolds increased SC collagen production compared to the plain scPLCL scaffolds. These characteristics make the A2P-releasing scPLCL scaffold highly interesting, with potential for vaginal tissue engineering and other applications benefiting from high collagen yield.

## Data Availability

The data presented in this study are available on request from all the authors.
